# 

**Published:** 1891-05

**Authors:** 


### CONTENTS:

Article I.—Maladies Associated with and Dependent upon Affections of the Teeth. By Rich'd Grady, M.D., D.D.S. 1

" II.—Effects of Sanguinary Deposits on the Teeth. By B. F. Arrington, D. D. S. - - - - 20

" III.—A Case Relating to the Influence of Maternal Impressions upon the Fœtus. By R. Osgood Mason, M. D. 22

" IV.—Chemistry, Physiology and Pathology of Nitrous Oxide—Practical Points, with Comments. By Dr. S. J. Hayes. - - - - - - - 27

" V.—The Education of Physicians and Dentists. By Prof. H. C. Wood. - - - - - - 31

" VI.—Extracting Teeth. By W. H. Sutton, L. D. S. - 33

" VII.—Resuscitation in Threatened Death from Chloroform. Prof. F. T. Miles. - - - - - - - 34

### EDITORIAL.

The American Dental Society of Europe. - - - - - 36

The American Dental Association. - - - - - - 39

Central Dental Association of Northern New Jersey.—Meeting of Committees on Dental Patents. - - - - - 40

Illinois State Dental Society. - - - - - - 41

Chicago Dental Society. - - - - - - - - 41

### MONTHLY SUMMARY.

Bleeding After Extraction. - - - - - - 41

The Detection of Abscess. - - - - - - 43

How to Keep Needles and Small Instruments from Rusting. - 44

How to Loosen Glass Stoppers. - - - - - 45

Risks of Cocaine Injections. - - - - - - 46

Simple Treatment of Ingrown Toenail. - - - - 46

Iodine in the Treatment of Warts. - - - - - 47

A Safe Local Anæsthetic. - - - - - - 47

Electricity in Extraction. - - - - - - - 48

Phosphate Fillings. - - - - - - - 48

Dark Joints. - - - - - - - - - 48

